# Emerging Evasion Mechanisms of Macrophage Defenses by Pathogenic Bacteria

**DOI:** 10.3389/fcimb.2020.577559

**Published:** 2020-09-25

**Authors:** Clarisse Leseigneur, Pierre Lê-Bury, Javier Pizarro-Cerdá, Olivier Dussurget

**Affiliations:** ^1^Unité de Recherche Yersinia, Institut Pasteur, Paris, France; ^2^Université de Paris, Sorbonne Paris Cité, Paris, France; ^3^National Reference Laboratory Plague & Other Yersiniosis, Institut Pasteur, Paris, France; ^4^WHO Collaborative Research & Reference Centre for Yersinia, Institut Pasteur, Paris, France

**Keywords:** phagocyte, immune escape, virulence, listeriosis, staphylococcal infection, plague, yersiniosis

## Abstract

Macrophages participate to the first line of defense against infectious agents. Microbial pathogens evolved sophisticated mechanisms to escape macrophage killing. Here, we review recent discoveries and emerging concepts on bacterial molecular strategies to subvert macrophage immune responses. We focus on the expanding number of fascinating subversive tools developed by *Listeria monocytogenes, Staphylococcus aureus*, and pathogenic *Yersinia* spp., illustrating diversity and commonality in mechanisms used by microorganisms with different pathogenic lifestyles.

## Introduction

**Figure 1:**
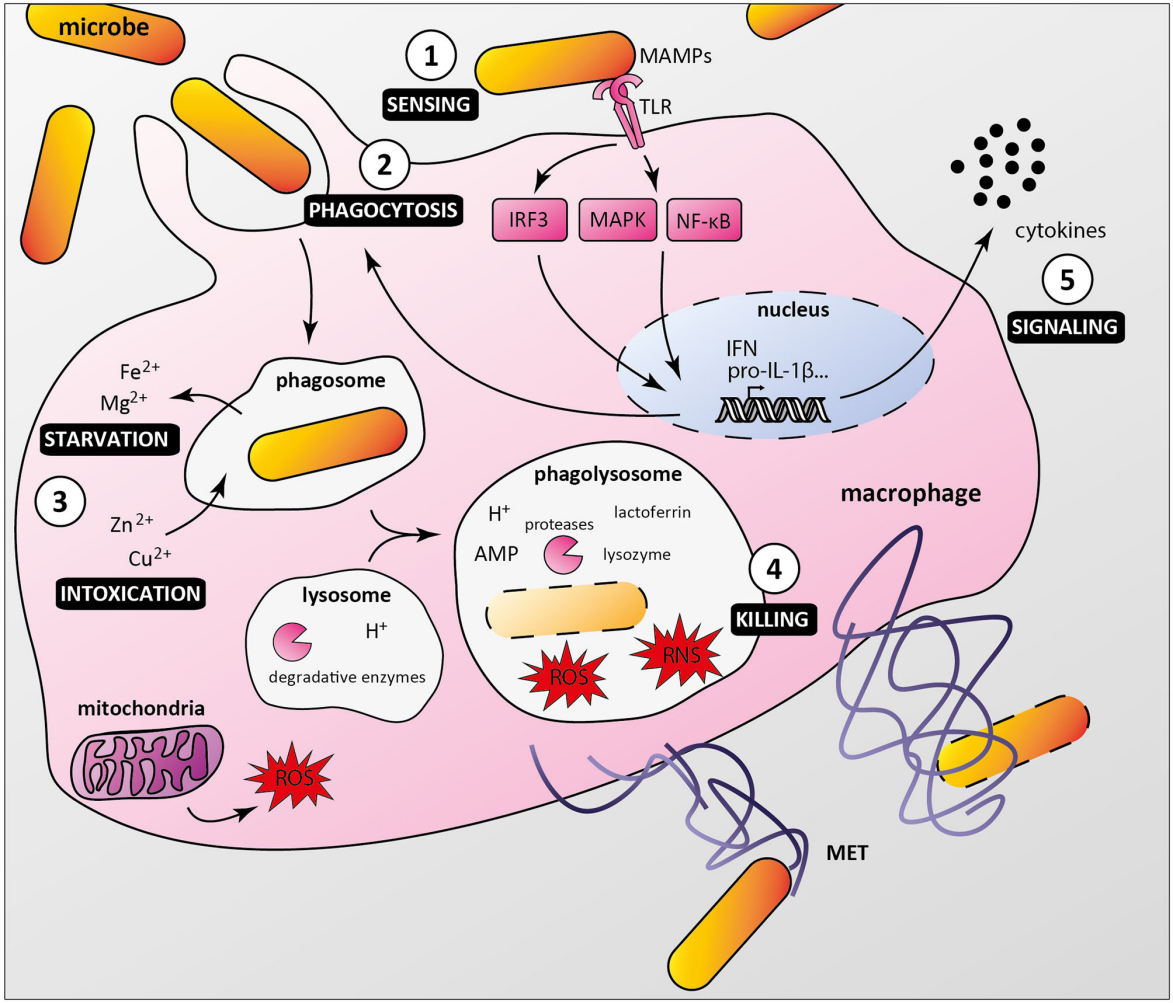
Macrophage anti-microbial mechanisms. **(1)** Bacteria are recognized by macrophage pattern recognition receptors (PRRs) such as Toll-like receptors (TLR), which bind conserved microbe-associated molecular patterns (MAMPs). **(2)** MAMP/PRR interaction triggers signaling cascades (e.g., IRF3, MAPKs, NF-κB) leading to macrophage responses, including formation of the phagocytic cup. **(3)** Internalized bacteria reside in phagosomes, from which nutrients and essential factors such as iron and magnesium are transported to the cytoplasm, restricting their supply to bacteria. Macrophages combine this starvation strategy with a poisoning mechanism involving phagosomal import of toxic amount of zinc and copper. **(4)** Phagosome maturation and fusion with lysosomes lead to acidification of the compartment lumen and activation of digestive enzymes such as proteases, which along with antimicrobial peptides (AMP), reactive oxygen and nitrogen species (ROS and RNS), lysozyme and lactoferrin contribute to bacterial killing. Macrophages can also undergo ETosis to release macrophage extracellular traps (MET) that immobilize and kill extracellular bacteria. **(5)** Additionally, infected macrophages secrete multiple cytokines to attract and activate other cells, which contribute to an effective immune response.

As professional phagocytes, macrophages are key components of host first line of defense against infection. Upon sensing local microenvironmental signals, macrophages display a continuous spectrum of functional characteristics, known as macrophage polarization, leading to microbicidal M1 or M2 macrophages associated with tissue repair and inflammation resolution (Locati et al., 2020). Macrophages detect pathogenic microorganisms by expressing pattern recognition receptors (PRRs), which interact with conserved microbe-associated molecular patterns (MAMPs). Among PRRs, Toll-like receptors (TLRs) play a major role in triggering immune responses as they recognize specifically a wide range of MAMPs, such as lipoproteins, lipopolysaccharide, flagellin, DNA, and RNA (Fitzgerald and Kagan, 2020). PRRs/MAMPs interactions activate signaling pathways, ultimately leading to cytokine production and/or phagocytosis (Figure 1). Once internalized, microorganisms are located in phagosomes, which mature and fuse with lysosomes, creating phagolysosomes. These acidic vesicles contain multiple antimicrobial molecules such as proteases, reactive oxygen species (ROS), reactive nitrogen species (RNS), and antimicrobial peptides, which contribute to degradation of pathogens (Levin et al., 2016). Macrophages also use nutritional immunity to actively sequester nutrients, thus preventing bacteria to acquire essential factors such as iron and manganese (Sheldon and Skaar, 2019). In addition, macrophages have been shown to produce macrophage extracellular traps that immobilize and kill pathogens (Doster et al., 2018).

Despite this powerful arsenal, macrophages fail to eliminate a wide variety of pathogens, which evolved complex strategies to counter and evade host immune system (Baxt et al., 2013). Some microorganisms prevent immune recognition by modulating their surface components, secrete immunomodulators to inhibit macrophage activation, hide in host cells or kill immune cells directly through toxin secretion and/or indirectly by inducing apoptosis (Kaufmann and Dorhoi, 2016). Others are able to evade phagocytosis and antigen presentation and highjack host cell pathways to acquire nutrients and ensure their survival (Hmama et al., 2015; Kaufmann and Dorhoi, 2016; Mitchell et al., 2016). While pathogens share multiple mechanisms, they developed specific evasion strategies depending on their pathogenic lifestyle. In this minireview, we will present recent advances in our understanding of macrophage subversion by important pathogenic bacteria characterized by specific life cycles: *Listeria monocytogenes, Staphylococcus aureus* and *Yersinia* spp.

## Listeria Monocytogenes

*Listeria monocytogenes* is the etiologic agent of listeriosis, a foodborne infection whose clinical manifestations range from self-limiting enteritis in immunocompetent individuals to life-threatening sepsis and meningo-encephalitis in the elderly and newborns. Three decades of research established this facultative intracellular bacterium as a model to study cellular and infection microbiology (Impens and Dussurget, 2020; Lecuit, 2020). *L. monocytogenes* fascinating life cycle in macrophages, i.e., entry, phagosomal escape, replication, actin-based movement and spread, was first described by Tilney and Portnoy (Tilney and Portnoy, 1989). This seminal study paved the way for identification of the major factors required to bypass cellular defenses and promote bacterial replication, including the pore-forming toxin listeriolysin O (LLO), PlcA and PlcB phospholipases, the ActA surface protein necessary for actin-based motility and their transcriptional activator PrfA (Radoshevich and Cossart, 2018).

**Figure 2:**
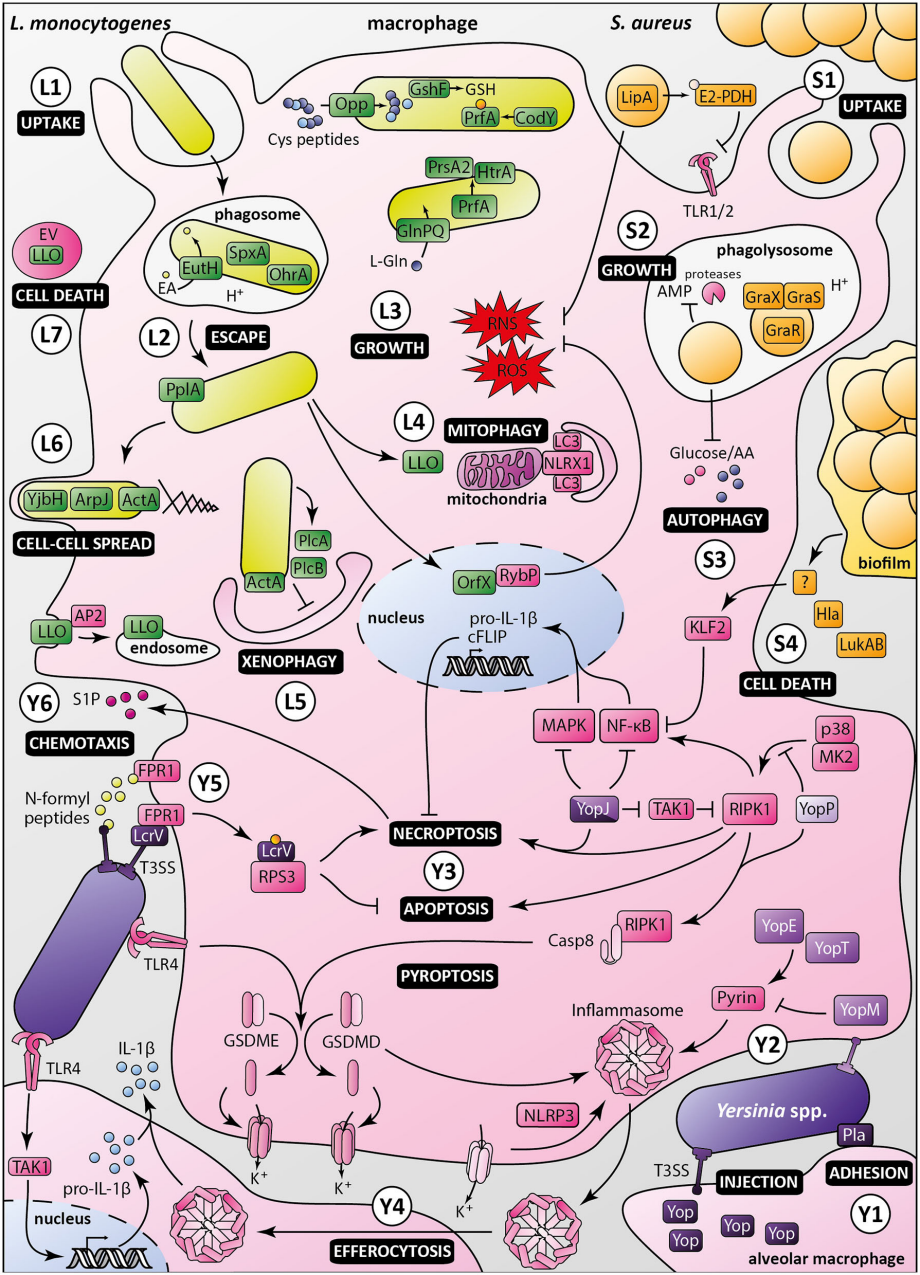
Macrophage evasion mechanisms by *Listeria monocytogenes, Staphylococcus aureus* and pathogenic *Yersinia. Listeria monocytogenes:* **(L1)** Upon uptake by the macrophage, *Listeria monocytogenes* is engulfed in a phagosome, in which ethanolamine uptake through EutH permease and activation of redox-responsive *spxA1* and *ohrA* are required for its survival. **(L2)** In addition to secretion of LLO and phospholipases, processing of the PlpA lipoprotein is required for phagosomal escape. **(L3)** Once in the cytosol, bacterial growth and virulence are mediated by the master regulator PrfA, which is activated by CodY and glutathione (GSH). The Opp permease ensures importation of cysteine-containing oligopeptides to allow glutathione synthesis by GshF. Full expression of *Listeria* virulence genes requires appropriate amounts of L-glutamine imported by the high-affinity ABC transporter GlnPQ. PrfA triggers secretion of PrsA2 chaperone and HtrA chaperone/protease, whose functions are required for invasion and intracellular growth. **(L4)** *Listeria* induces mitophagy through the oligomerization of NLRX1 receptor by LLO, lowering ROS levels, and promoting bacterial survival. *Listeria* also controls ROS levels by secretion of the nucleomodulin OrfX, which interacts with the regulator RybP. **(L5)** ActA inhibits xenophagy along with PlcA and PlcB, which block LC3 lipidation. *Listeria* intracellular survival also depends on Ap2a2-mediated control of LLO cytotoxicity by restricting its cytosolic activity. **(L6)** Besides ActA, YjbH, and ArpJ are required for efficient bacterial spread from cell to cell. **(L7)** *Listeria* produces extracellular vesicles (EV) containing many virulence factors, including LLO, to promote macrophage death and control innate immunity response. *Staphylococcus aureus:* **(S1)** *Staphylococcus aureus* is phagocytosed by macrophages. The moonlighting metabolic protein pyruvate dehydrogenase, once lipoylated by LipA, suppresses macrophage activation by lipopeptides through binding to TLR1/2. LipA also decreases RONS production. **(S2)** Staphylococci reside and multiply in mature phagosomes, through sensing of acidification by GraXRS and activation of several genes allowing bacterial replication and resistance to antimicrobial peptides (AMP). **(S3)** *S. aureus* also modulates metabolic fluxes to induce a starvation-like state of macrophages, triggering autophagy. **(S4)** Secretion of alpha-toxin Hla and leukocidin AB (LukAB), besides having a direct cytotoxic effect, inhibits macrophage phagocytosis and promotes biofilm formation. Through a yet unknown intermediate, biofilm conditioned medium attenuates NF-κB activation by increasing KLF2 expression. Pathogenic *Yersinia*, i.e., *Y. enterocolitica* (light purple), *Y. pseudotuberculosis* (purple), and *Y. pestis* (dark purple): **(Y1)** In the lungs, *Yersinia* adheres to alveolar macrophages through Pla, which shows immunosuppressive properties. **(Y2)** Injection of Yops, virulence effectors, in macrophages through the T3SS allows manipulation of host cell pathways. In absence of YopM, YopE, and YopT activate the inflammasome by dephosphorylating pyrin. **(Y3)** Once translocated into the host cell, LcrV is glutathionylated, promoting binding to RPS3, suppressing apoptosis and increasing necroptosis. YopJ inhibition of TAK1 leads to activation of RIPK1 and induction of necroptosis or apoptosis of targeted macrophage. YopJ also inhibits MAPK and NF-κB pathways, inhibiting pro-IL-1β production and limiting pro-inflammatory response. YopP inhibits RIPK1 phosphorylation by p38^MAPK^/MK2, triggering macrophage apoptosis and activation of cell death effectors gasdermin D and E. **(Y4)** Macrophages intoxicated with low levels of YopJ can release IL-1β upon uptake of inflammasome from highly intoxicated dead cells, possibly by efferocytosis. **(Y5)** N-formylpeptides released by *Y. pestis* are recognized by host receptor FPR1, promoting immune cell chemotaxis toward bacteria. Adhesion of bacteria to macrophage is mediated by FPR1/LcrV interaction, which allows the assembly of type three secretion system. **(Y6)** Sphingosine-1-phosphate released from dead cells attracts new phagocytes, which in turn are targeted by *Yersinia* released from necroptotic cells, ultimately promoting infection.

PrfA is the master regulator of virulence in *L. monocytogenes*. New facets of its properties have recently been revealed (Figure 2). PrfA was shown to induce secretion of the chaperone PrsA2 and the chaperone/protease HtrA, whose protein folding and stabilizing functions promote bacterial fitness and survival during infection of macrophages (Ahmed and Freitag, 2016). In addition, PrfA function has been reported to depend on the balance between activating and inhibitory oligopeptides imported by the Opp permease (Krypotou et al., 2019). Cysteine-containing peptides provides cysteine necessary for synthesis of glutathione, the PrfA activator, contributing to *L. monocytogenes* survival in macrophages. This study uncovers a new mechanism of regulation of PrfA by controlling the oligopeptide composition of the environment. It also reinforces the link between metabolism and virulence previously underscored by the demonstration of PrfA activation by the global nutritional regulator CodY (Lobel et al., 2015). Along the same lines, L-glutamine imported by the GlnPQ ABC transporter, has been shown to be an indicator of intracellular localization and an inducer of *L. monocytogenes* virulence genes (Haber et al., 2017).

One of the most important virulence factors positively regulated by PrfA is LLO. This cholesterol-dependent cytolysin forms pores in the phagosome membrane, resulting in vacuolar rupture and bacterial escape to the cytosol. However, it is expressed at all stages of the intracellular cycle and could be cytotoxic if active outside of the phagosome. The N-terminal PEST-like sequence of LLO is essential to restrict its cytosolic activity and prevent cell killing (Decatur and Portnoy, 2000). The adaptor-related protein complex 2 Ap2a2, a subunit of the AP-2 endocytic machinery, has recently been shown to interact with the PEST-like region of LLO, revealing how cytotoxicity is controlled (Chen et al., 2018). Recognition of LLO by AP-2 triggers its endocytic removal from plasma membrane and its degradation, possibly through autophagosomal or multivesicular body-mediated pathways. The acidic content of the phagosome is known to be optimal for LLO, which contributes to the compartmentalization of its activity. The transient exposure of *L. monocytogenes* to the low pH of the phagosome imposes mechanisms of adaptation. The ethanolamine permease EutH is required for ethanolamine uptake and promotes bacterial growth at low pH *in vitro*. Anderson et al. broadened this concept by showing that EutH is important for *L. monocytogenes* survival in the phagosome (Anderson et al., 2018).

Additional new genes involved in the intracellular life cycle of *L. monocytogenes* have been identified by the Portnoy's lab using an elegant strategy relying on screening a library of *himar1* transposon mutants constructed in a Cre/Lox-based suicide strain that failed to replicate in macrophages upon activation of ActA (Reniere et al., 2016). The *spxA1* gene encoding a putative disulfide stress transcriptional regulator and the *ohrA* gene encoding a peroxiredoxin domain-containing protein of the organic hydroperoxide resistance subfamily, were required for *L. monocytogenes* survival in the phagosome and optimal replication in bone marrow-derived macrophages. The *yjbH* gene encoding a putative thioredoxin and the *arpJ* gene encoding an amino-acid permease were required for *L. monocytogenes* spread from cell-to-cell. This study confirmed the contribution of the PplA lipoprotein to *L. monocytogenes* intracellular life cycle. Processing of PplA leads to secretion of a peptide, which has been previously shown to be required for vacuolar escape (Xayarath et al., 2015). *L. monocytogenes* glutathione synthase gene *gshF* was expectedly identified in the screen, as glutathione is an allosteric activator of PrfA (Reniere et al., 2015). Overall, these findings point to an important role of redox metabolism during *L. monocytogenes*/macrophages interactions.

After rupture of the phagosomal membrane, bacteria replicate in the cytosol. Our understanding of the mechanisms by which *L. monocytogenes* evades cell defenses and survives intracellularly has significantly improved in the last 5 years. Besides its spectacular role in actin-based propulsion of bacteria, ActA has long been known to play a key role in escape from autophagic recognition by recruitment of the Arp2/3 complex and Ena/VASP at the bacterial surface (Birmingham et al., 2007; Yoshikawa et al., 2009). Several studies confirmed the importance of ActA and the role of PlcA and PlcB in escape from autophagy (Tattoli et al., 2013; Mitchell et al., 2015, 2018). In addition to their contribution to vacuolar rupture, phospholipases are required for bacterial multiplication in infected cells by subverting the autophagic process, possibly by blocking LC3 lipidation. *Listeria monocytogenes* also escapes innate immune response by inducing mitophagy in macrophages (Zhang et al., 2019). Secretion of LLO triggers oligomerization of the mitophagy receptor NLRX1, resulting in increased mitophagy, lower levels of mitochondrial ROS and increased bacterial survival. Another mechanism evolved by *L. monocytogenes* to evade macrophage oxidative defenses is secretion of the nucleomodulin OrfX (Prokop et al., 2017). This PrfA-regulated virulence factor has been shown to inhibit ROS and NO production in infected macrophages. OrfX is targeted to the nucleus and interacts with RybP, a regulatory protein that controls infection. OrfX decreases RybP levels, thereby promoting bacterial survival.

*Listeria monocytogenes* also subverts cellular processes by producing extracellular vesicles. Coelho et al. demonstrated that *L. monocytogenes* secretes vesicles that contain many virulence factors, including LLO and PlcA (Coelho et al., 2019). These vesicles mediate LLO-dependent macrophage toxicity, consolidating the emerging concept of extracellular vesicles as prominent weapons in host-pathogen interactions.

## Staphylococcus aureus

*Staphylococcus aureus* is a highly successful facultative intracellular opportunistic pathogen, which has developed many mechanisms to counter host immune defenses. This arsenal allows bacteria to infect virtually any human tissue, leading to diverse clinical manifestations, ranging from mild to severe skin and soft tissue infections to life-threatening endocarditis, necrotizing pneumonia or septicemia (Tong et al., 2015). *S. aureus* secretes several toxins that can directly and specifically interfere with cellular functions, trigger cell death or damage immune cells (Spaan et al., 2017). This pathogen is also able to withstand phagocyte-mediated killing. Though most studies on *S. aureus*/phagocyte interactions focused on neutrophils (de Jong et al., 2019), macrophages also gained attention as they may be “Trojan horses” used by bacteria to disseminate throughout the body. Accordingly, *S. aureus* can resist phagocytic oxidative and nitrosative killing, antimicrobial peptides and nutritional immunity (Flannagan et al., 2015). Here, we present recent findings uncovering novel mechanisms developed by *S. aureus* to evade macrophage-mediated killing (Figure 2).

Contrary to *L. monocytogenes*, which quickly escapes phagosomes and replicates in the cytosol, *S. aureus* resides and multiplies in mature phagolysosomes in murine and human macrophages (Flannagan et al., 2016). Bacteria divide in mature phagolysosomes and trigger macrophage death by apoptosis or necroptosis rather than membrane disruption. Acidification of the phagolysosome, usually a bactericidal mechanism, is sensed by *S. aureus* through the GraXRS regulatory system (Flannagan et al., 2018). It triggers transcription of genes involved in adaptation to the phagolysosome hostile environment such as resistance to antimicrobial peptides. This adaptation is independent of toxin production, as mutants of the Agr quorum sensing system or SaeR, two major regulators of toxin production, and alpha phenol soluble modulin mutants can still replicate in macrophages. Interestingly, this seems in contradiction with previous reports showing that *agr* expression was induced in THP1 macrophages and by acidic pH (Tranchemontagne et al., 2015). Strains, cell lines and methodological differences could explain discrepancies between these studies, as discussed by Flannagan et al. (2018). Multiplication of *S. aureus* in mature phagolysosomes, ultimately leading to cell death and bacterial dissemination, is compatible with the “Trojan horse” hypothesis.

The link between metabolism, immunity and virulence has recently gained attention. Once inside macrophages, *S. aureus* exploits host cell metabolism to ensure its own proliferation. Methicillin-resistant *S. aureus* infection of HeLa cells and bone marrow derived macrophages from BALB/c mice has been shown to modulate cellular metabolic fluxes, depleting notably glucose and amino acid pools (Bravo-Santano et al., 2018). These changes induce a starvation-like state of infected cells, activating AMPK and ERK pathways and triggering autophagy. Inhibition of autophagy blocked *S. aureus* replication, suggesting that metabolic activation of autophagy is essential for intracellular bacterial proliferation. Proteins involved in bacterial metabolism may also play a role in immune evasion, as revealed by the study of moonlighting proteins (Wang et al., 2014). These multifunctional proteins are highly conserved in bacteria. In addition to their cytoplasmic role in metabolism and stress response, some of them are released outside of bacteria where they contribute to virulence properties such as tissue adhesion and immune escape. For instance, *S. aureus* can blunt macrophage activation by modification and secretion of the moonlighting protein pyruvate dehydrogenase (PDH) (Grayczyk et al., 2017). The authors showed in this study that bacterial lipoic acid synthetase LipA adds a lipoic acid on the E2 subunit of the PDH complex, yielding lipoyl-E2-PDH whose role in the cytoplasm is to convert pyruvate to acetyl-CoA. The lipoyl-E2-PDH is excreted by bacteria and suppresses macrophage activation through specific binding and inhibition of TLR1/2 heterodimer activation. The same group also showed that LipA decreases production of ROS and reactive nitrogen species (RNS) by NADPH oxidase and iNOS, respectively, both *in vitro* and in a mouse model of *S. aureus* infection (Grayczyk and Alonzo, 2019). Together, these studies support the concept that specific cytoplasmic proteins moonlight outside bacteria and link metabolism and virulence.

*Staphylococcus aureus* capacity to form biofilms is important for successful immune escape. A biofilm matrix surrounding bacteria prevents recognition and phagocytosis by immune cells. In addition, biofilms may directly dampen immune response by polarizing macrophages toward an anti-inflammatory phenotype (Thurlow et al., 2011). More recently, it was shown that *S. aureus* growing in biofilms subverts immune responses through active secretion of virulence factors (Scherr et al., 2015). The authors demonstrated that alpha-toxin Hla and leukocidin LukAB have a synergistic action resulting in inhibition of macrophage phagocytosis and induction of cytotoxicity, promoting biofilm formation in a murine model of *S. aureus* orthopedic implant infection. Another group showed that biofilm-conditioned medium was responsible for attenuated NF-κB activation due to increased expression of the anti-inflammatory transcription factor Kruppel-like factor 2 in RAW264.7 macrophages (Alboslemy et al., 2019). While bacterial molecule(s) responsible for KLF2 induction remain(s) to be identified, these findings confirm the importance of virulence factor secretion to suppress macrophage functions in addition to the role of biofilms as a physical barrier.

## Yersinia

The *Yersinia* genus includes three human pathogenic species: the two enteropathogens *Yersinia enterocolitica* and *Yersinia pseudotuberculosis*, as well as *Yersinia pestis*, the etiological agent of plague. The three species harbor the pCD1 plasmid coding for a type three secretion system (T3SS) and *Yersinia* outer proteins Yops, potent virulence effectors that are injected in target cells. In contrast to *L. monocytogenes* and *S. aureus*, pathogenic *Yersinia* are well-equipped to manipulate host cells from the outside, by mechanisms recently reviewed (Pinaud et al., 2018; Demeure et al., 2019). Translocation of Yops into host immune cells modulates pyroptosis/apoptosis. Apoptosis is triggered via caspase-8 activation by YopP/J, while YopM and YpkA activate caspase-3. Pyroptosis can be regulated by Yops acting on caspase-4/5, pyrin, NLRP3, ASC, RhoGTP or caspase-8. YopP/J and YopE also interfere with MAPK and NF-kB signaling. Depending on the phase of the disease, *Yersinia* spp. trigger pro- or anti-inflammatory responses. Yops can also prevent phagocytosis: YopH, YopT, YopE, and YpkA/YopO can block phagocytosis signaling and modulate GTPases or actin. In addition to Yops, capsular antigen fraction 1 (F1) and Psa fimbria (pH 6 antigen) prevent macrophage adhesion and phagocytosis. Psa and Ail outer-membrane protein enhance Yops delivery, and Ail and Pla outer-membrane protein promote cell attachment and invasion.

Recent studies refined our understanding of *Yersinia* interactions with macrophages, in particular subversion of cell death processes (Figure 2). *Y. enterocolitica* YopP has been shown to inhibit phosphorylation of the master regulator of cell fate RIPK1 by p38^MAPK^/MK2 (Menon et al., 2017). Upon suppression of MK2 activity, RIPK1 autophosphorylates and triggers macrophage apoptosis to promote infection. Along the same lines, in a murine model of bubonic plague, *Y. pestis* YopJ induced RIPK1-dependent necroptotic cell death causing bubo necrosis. Necroptosis was delayed by pro-survival factors such as FLIP, allowing *Y. pestis* to replicate inside macrophages before cell death. Sphingosine-1-phosphate secreted by dying cells attracted new phagocytes, which could be infected by bacilli released from necroptotic cells, ultimately enhancing spread of *Y. pestis* (Arifuzzaman et al., 2018). Gasdermin D (GSDMD) and gasdermin E (GSDME) are cell death effectors triggering cell membrane permeabilization and potassium efflux upon cleavage by caspase-1/4/11 and caspase-3/7, respectively. Two studies recently reported an additional pathway controlling gasdermin processing. In murine macrophages infected with *Y. pseudotuberculosis*, YopJ induced cell death by inhibiting TAK1 and by RIPK1/caspase-8-dependent cleavage of gasdermin D (GSDMD) and gasdermin E (GSDME) downstream of TLR4-TRIF activation (Orning et al., 2018; Sarhan et al., 2018). Orning et al. suggest that *Yersinia* spp. inhibition of TAK1 results in cell death with features of both apoptosis and pyroptosis and in GSMD-dependent activation of the NLRP3 inflammasome leading to IL-1β release. Sarhan et al. hypothesized that IL-1β production requires an heterogeneous population of infected macrophages. Macrophages with levels of YopJ too low to block MAPK signaling retain their capacity to produce pro-IL-1β which is then possibly matured by efferocytosis of dead cells, or uptake of ASC/NLRP3 inflammasomes released from dead cells that were heavily intoxicated. Interestingly, human macrophages are resistant to cell death induced by TAK1 inhibition and produced little if any IL-1β (Sarhan et al., 2018). This resistance and TLR4 hypo-responsiveness to *Y. pestis* tetra-acylated LPS presumably contribute to the initially silent preinflammatory phase of plague. Macrophage infection with *Yersinia* spp. can trigger activation of the NLRP3 inflammasome but also caspase-1 inflammasome assembled upon RhoA-sensitive pyrin activation (Orning et al., 2018; Medici et al., 2019). RhoA-targeting YopE and YopT trigger inflammasome assembly, in absence of YopM that inactivates pyrin. In *Y. pseudotuberculosis*, YopE and, to a lower extent YopT, were recently shown to induce dephosphorylation of pyrin Ser205, thereby activating the inflammasome (Medici et al., 2019). This study reveals that RhoA specificity of Yops affects pyrin activation.

Selective destruction of immune cells by *Y. pestis* is a landmark of plague, allowing bacterial multiplication and systemic spread. The plague receptor on human immune cells has recently been identified by the Schneewind's group (Osei-Owusu et al., 2019). A CRISPR-Cas9 screen in U937 macrophages linked *Y. pestis* T3SS-mediated killing to N-formylpeptide receptor FPR1, a member of the GPCR family that triggers immune cells chemotaxis and cytokine production upon sensing N-formylpeptides released by bacteria. Binding of the T3SS cap protein LcrV to FPR1 was necessary for assembly of the translocon and injection of effectors, which subvert signaling pathways and trigger cell death. Of note, FPR1 is not essential for *Y. pestis* effector translocation in mouse macrophages. Importantly, this study identified human *FRP1 R190W* as a potential plague resistance allele. The same group discovered that *Y. pestis* LcrV was glutathionylated at Cys273 with host-derived glutathione, a posttranslational modification required for successful infection in mice and rats (Mitchell et al., 2017). Macrophage ribosomal protein S3 (RPS3) was identified as a ligand of LcrV. RPS3 is a component of ribosomal 40S subunit and a regulator of apoptosis, DNA repair and innate immune response. Glutathionylation of LcrV promoted RPS3 binding, reduced the rate of effector injection, suppressing apoptosis, increasing necroptosis and IL-1β and IL-18 release, triggering an inflammatory response correlated with increased virulence.

An early preinflammatory phase is critical for *Y. pestis* proliferation in lungs and progression of pneumonic plague. While importance of Yops in this early phase has long been known, a recent report refined the role of the plasminogen activator protease Pla in the progression of primary pneumonic plague (Banerjee et al., 2019). Using human precision-cut lung slices, human primary alveolar macrophages and a murine intranasal infection model, the authors showed that Pla was required for bacterial adherence and optimal effector secretion into alveolar macrophages, the primary host cells targeted by *Y. pestis* in the early phase of pneumonic plague. They further showed that Pla contributes to dampen inflammatory cytokine production in the human lung model, establishing its immunosuppressive properties and supporting its role in the preinflammatory phase of pneumonic plague.

## Perspectives

*L. monocytogenes, S. aureus*, and *Yersinia* spp. have been powerful model organisms to decipher the molecular mechanisms of interactions between pathogenic bacteria and macrophages. While the universe of bacterial subversion strategies has recently expanded, our understanding of macrophage biology has also immensely progressed (Ginhoux and Guilliams, 2016; Gao et al., 2017; Gordon and Plüddemann, 2019; Guilliams et al., 2020) and pathogenomics data showing within-species heterogeneity skyrocketed (Bosi et al., 2016; Maury et al., 2016; Seif et al., 2018; Savin et al., 2019; Oyas et al., 2020), uncovering an unforeseen complexity. Future studies of the dialogue between bacteria and these cells will thus be more challenging than ever and will undoubtedly rely on technological developments, in particular in imaging and systems biology. In parallel, refinement of cellular, tissular and *in vivo* infection models will be required in order to discover relevant new concepts. In particular, heterogeneity of bacterial and macrophage populations, spatiotemporal dynamics of interactions and multiplicity of microenvironmental cues can hardly be recapitulated in standard assays using cell lines. This calls for appropriate *in vivo* infection models, which frequently reveals unexpected findings that were not or could not be observed *in vitro* (Blériot et al., 2015; Jones and D'Orazio, 2017; Gluschko et al., 2018; Paudel et al., 2020) and which are instrumental to development of innovative therapeutic strategies (Dickey et al., 2017; Kaufmann et al., 2018; Morrison, 2020).

## Author Contributions

All authors listed have made a substantial, direct and intellectual contribution to the work, and approved it for publication.

## Conflict of Interest

The authors declare that the research was conducted in the absence of any commercial or financial relationships that could be construed as a potential conflict of interest.
